# *N*-[Bis(benzylamino)phosphoryl]-2,2,2-trichloroacetamide

**DOI:** 10.1107/S1600536810017873

**Published:** 2010-05-22

**Authors:** Vladimir A. Ovchynnikov

**Affiliations:** a National Taras Shevchenko University, Department of Chemistry, Volodymyrska str. 64, 01033 Kyiv, Ukraine

## Abstract

In the title compound, C_16_H_17_Cl_3_N_3_O_2_P, the P atom has a slightly distorted tetra­hedral configuration. The conformations of the carbonyl and phosphoryl groups are *anti* to each other. In the crystal, inter­molecular N—H⋯O hydrogen bonds link the mol­ecules into infinite chains parallel to the *b* axis.

## Related literature

For the use of carbacyl­amido­phosphates as potential new ligands, see: Skopenko *et al.* (1996 ▶); Ovchynnikov *et al.* (1998 ▶); Znovjak *et al.* (2009 ▶); Gubina *et al.* (2009 ▶); Gowda *et al.* (2010 ▶); Amirkhanov *et al.* (1997*a* ▶); Safin *et al.* (2009 ▶). For their biological activity, see: Amirkhanov *et al.* (1996 ▶); Rebrova *et al.* (1982 ▶). For P=O bond lengths, see: Amirkhanov *et al.* (1997*b* ▶). For the synthesis of the title compound, see: Kirsanov & Derkach (1956 ▶). 
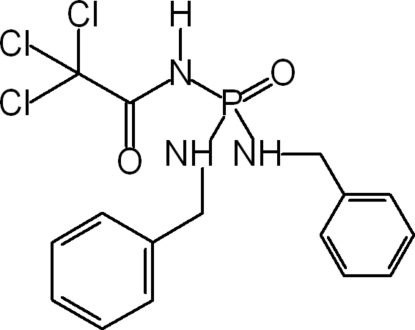


## Experimental

### 

#### Crystal data

C_16_H_17_Cl_3_N_3_O_2_P
*M* *_r_* = 420.65
Triclinic, 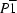

*a* = 9.116 Å
*b* = 10.3586 (2) Å
*c* = 11.7713 (2) Å
α = 68.320 (1)°
β = 67.762 (1)°
γ = 86.469 (1)°
*V* = 952.13 (3) Å^3^
*Z* = 2
Mo *K*α radiation
μ = 0.58 mm^−1^
*T* = 293 K
0.20 × 0.20 × 0.20 mm

#### Data collection

Oxford Diffraction Xcalibur3 diffractometer
Absorption correction: multi-scan (*CrysAlis RED*; Oxford Diffraction, 2009 ▶) *T*_min_ = 0.893, *T*_max_ = 0.893
4976 measured reflections
3286 independent reflections
3069 reflections with *I* > 2σ(*I*)
*R*_int_ = 0.016

#### Refinement

*R*[*F* ^2^ > 2σ(*F* ^2^)] = 0.038
*wR*(*F* ^2^) = 0.103
*S* = 1.05
3286 reflections
226 parameters
H-atom parameters constrained
Δρ_max_ = 0.62 e Å^−3^
Δρ_min_ = −0.41 e Å^−3^

### 

Data collection: *CrysAlis CCD* (Oxford Diffraction, 2009 ▶); cell refinement: *CrysAlis RED* (Oxford Diffraction, 2009 ▶); data reduction: *CrysAlis RED*; program(s) used to solve structure: *SHELXS97* (Sheldrick, 2008 ▶); program(s) used to refine structure: *SHELXL97* (Sheldrick, 2008 ▶); molecular graphics: *ORTEPIII* (Burnett & Johnson, 1996 ▶), *ORTEP-3 for Windows* (Farrugia, 1997 ▶) and *PLATON* (Spek, 2009 ▶); software used to prepare material for publication: *SHELXL97*.

## Supplementary Material

Crystal structure: contains datablocks I, global. DOI: 10.1107/S1600536810017873/dn2554sup1.cif

Structure factors: contains datablocks I. DOI: 10.1107/S1600536810017873/dn2554Isup2.hkl

Additional supplementary materials:  crystallographic information; 3D view; checkCIF report

## Figures and Tables

**Table 1:** Hydrogen-bond geometry (Å, °)

*D*—H⋯*A*	*D*—H	H⋯*A*	*D*⋯*A*	*D*—H⋯*A*
N1—H1⋯O1^i^	0.86	1.95	2.790 (2)	167
N2—H2⋯O2^ii^	0.86	2.23	3.055 (2)	161

Symmetry codes: (i) 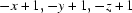; (ii) 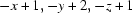.

**Fig. 1.**
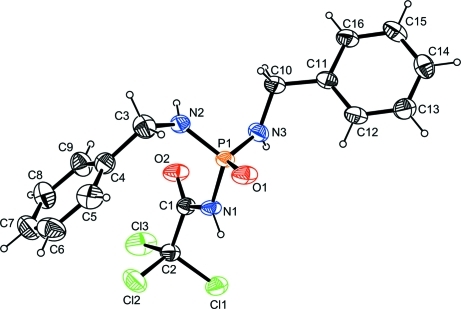
Molecular structure of the title compound with the atom labeling scheme. Ellipsoids are drawn at the 50% probability level. H atoms are represented as small spheres of arbitrary radii.

**Fig. 2.**
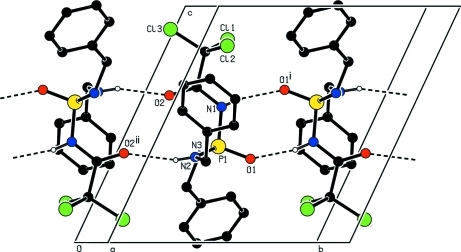
Partial packing view showing the hydrogen bonds pattern. Hydrogen bonds are shown as dashed lines. H atoms not involved in hydrogen bondings have been omitted for clarity. [Symmetry codes: (i) -x+1, -y+1, -z+1; (ii) -x+1, -y+2, -z+1]

## supplementary crystallographic information

### Comment

Carbacylamidophosphates of the general formula RC(O)NHP(O)R_2_ are potential new ligands for metal ions (Skopenko *et al.*, 1996; Ovchynnikov *et al.*, 1998; Znovjak *et al.*, 2009; Gubina *et al.*, 2009; Amirkhanov *et al.*, 1997a; Gowda *et al.*, 2010; Safin *et al.*, 2009). Many of these compounds also show biological activity, including anticancer activity (Amirkhanov *et al.*, 1996; Rebrova *et al.*, 1982). This work reports the structure of *N*,*N*'-dibenzyl-*N*"-trichloroacetylphosphortriamide (HDBA).

In the title compound, the phosphorus atom has a slightly distorted tetrahedral configuration (Fig.1). The average values of the angles OPN in the molecule are larger than tetrahedral, while the N – P – N angles are smaller, with the exception O1 – P1– N1 106.85 (7) ° and N(1) – P1– N(2) 112.32 (7) °, which can be rationalized by the influence of the hydrogen bonds. The environment of the nitrogen atoms is practically planar with only slight deviations from the mean planes.

The bond length P ═O (1.479 (1) Å is longer than in the compounds with alkyl amide substituents (the range of bond length d_(P __═__O)_ 1.475 - 1.478 Å) (Amirkhanov *et al.*, 1997b). In the structure the carbonyl and phosphoryl groups are *anti* to each other as in most carbacylamidophosphates.

The fragment including the atoms O(2), N(1), C(1), C(2) is virtually planar, with only slight deviations from the mean plane. The phosphorus and oxygen atom of the phosphoryl group do not fit into this plane. Close enough to this plane lie the hydrogen H(1 N) and one of the chlorine atoms Cl(3). The carbonyl oxygen-phosphorus distance 3.023 (1) Å is considerably shorter than the sum of Van der Waals radii (3.3 Å).

Molecules are linked by hydrogen bonds of the phosphorylic oxygen atoms and the hydrogen atoms of the C(O)N(H)P(O) groups of neighboring molecules. The N—H···O intermolecular hydrogen bonds pack the molecules into infinite chains parallel to the b axis (Table 1, Fig.2).

### Experimental

The solution of benzylamine (26.8 g, 0.25 mol) in 30 ml of chloroform was cooled to 10 °C and a solution of the dichloride of trichloroacetylamidophosphoric acid (14 g, 0.047 mol) in 150 ml of chloroform was added slowly with stirring. The temperature was not allowed to rise above 15 °C. Stirring was continued for about 40 min. The resulting mixture, containing HL, H_2_NCH_2_C_6_H_5_*HCl and excess dibenzylamine, was filtered from the precipitate (H_2_NCH_2_C_6_H_5_*HCl). Then the solution was evaporated and the residue was treated with aqueous HCl; the product precipitated as a yellow crystalline powder (90 % yield) (Kirsanov & Derkach, 1956). A colourless crystalline compound was obtained after recristallization from acetone. The compound is air stable, soluble in alcohols and hot acetone, insoluble in non-polar aprotic solvents and water, M.p. = 144 °C. *Anal.* Calc.: C 45.68,H 4.07, N 9.99; Found: C 45.73, H 3.95, N 9.85. IR (KBr pellet, cm^-1^): 1709 (s, CO) and 1250 (s, PO).

### Refinement

All hydrogen atoms were located from electron density difference maps and included in the refinement in the riding motion approximation with U_iso_ constrained to be 1.2 times U_eq_ of the carrier atom.

### Crystal data

**Table:**

C_16_H_17_Cl_3_N_3_O_2_P	*Z* = 2
*M**_r_* = 420.65	*F*(000) = 432
Triclinic, *P*1	*D*_x_ = 1.467 Mg m^−^^3^
Hall symbol: -P 1	Melting point: 417 K
*a* = 9.116 Å	Mo *K*α radiation, λ = 0.71073 Å
*b* = 10.3586 (2) Å	Cell parameters from 3594 reflections
*c* = 11.7713 (2) Å	θ = 2.0–27.1°
α = 68.320 (1)°	µ = 0.58 mm^−^^1^
β = 67.762 (1)°	*T* = 293 K
γ = 86.469 (1)°	Block, colorless
*V* = 952.13 (3) Å^3^	0.20 × 0.20 × 0.20 mm

### Data collection

**Table:**

Oxford Diffraction Xcalibur3 diffractometer	3286 independent reflections
Radiation source: Enhance (Mo) X-ray Source	3069 reflections with *I* > 2σ(*I*)
graphite	*R*_int_ = 0.016
Detector resolution: 16.1827 pixels mm^-1^	θ_max_ = 25.0°, θ_min_ = 2.0°
ω scans	*h* = −8→10
Absorption correction: multi-scan (*CrysAlis RED*; Oxford Diffraction, 2009)	*k* = −10→12
*T*_min_ = 0.893, *T*_max_ = 0.893	*l* = −13→13
4976 measured reflections	

### Refinement

**Table:**

Refinement on *F*^2^	Primary atom site location: structure-invariant direct methods
Least-squares matrix: full	Secondary atom site location: difference Fourier map
*R*[*F*^2^ > 2σ(*F*^2^)] = 0.038	Hydrogen site location: inferred from neighbouring sites
*wR*(*F*^2^) = 0.103	H-atom parameters constrained
*S* = 1.05	*w* = 1/[σ^2^(*F*_o_^2^) + (0.0496*P*)^2^ + 0.7601*P*] where *P* = (*F*_o_^2^ + 2*F*_c_^2^)/3
3286 reflections	(Δ/σ)_max_ < 0.001
226 parameters	Δρ_max_ = 0.62 e Å^−^^3^
0 restraints	Δρ_min_ = −0.40 e Å^−^^3^

### Special details

**Table:**

Experimental. CrysAlis RED, (Oxford Diffraction Ltd., 2007) Empirical absorption correction using spherical harmonics, implemented in SCALE3 ABSPACK scaling algorithm.
Geometry. All esds (except the esd in the dihedral angle between two l.s. planes) are estimated using the full covariance matrix. The cell esds are taken into account individually in the estimation of esds in distances, angles and torsion angles; correlations between esds in cell parameters are only used when they are defined by crystal symmetry. An approximate (isotropic) treatment of cell esds is used for estimating esds involving l.s. planes.
Refinement. Refinement of *F*^2^ against ALL reflections. The weighted *R*-factor wR and goodness of fit *S* are based on *F*^2^, conventional *R*-factors *R* are based on *F*, with *F* set to zero for negative *F*^2^. The threshold expression of *F*^2^ > σ(*F*^2^) is used only for calculating *R*-factors(gt) etc. and is not relevant to the choice of reflections for refinement. *R*-factors based on *F*^2^ are statistically about twice as large as those based on *F*, and *R*- factors based on ALL data will be even larger.

### Fractional atomic coordinates and isotropic or equivalent isotropic displacement parameters (Å^2^)

**Table:**

	*x*	*y*	*z*	*U*_iso_*/*U*_eq_	
Cl1	0.82223 (7)	0.78475 (7)	0.14155 (6)	0.04467 (17)	
Cl2	0.49492 (8)	0.81971 (8)	0.16751 (6)	0.0545 (2)	
Cl3	0.70636 (11)	1.05737 (6)	0.09763 (7)	0.0681 (3)	
P1	0.47348 (6)	0.66397 (5)	0.59367 (5)	0.02713 (15)	
O1	0.4132 (2)	0.51582 (15)	0.64581 (15)	0.0401 (4)	
O2	0.6029 (2)	0.94775 (15)	0.37756 (16)	0.0434 (4)	
N1	0.5631 (2)	0.71538 (17)	0.42584 (16)	0.0305 (4)	
H1	0.5828	0.6523	0.3921	0.037*	
N2	0.3308 (2)	0.75943 (18)	0.63801 (17)	0.0304 (4)	
H2	0.3531	0.8309	0.6511	0.036*	
N3	0.6122 (2)	0.7072 (2)	0.63210 (18)	0.0362 (4)	
H3	0.7059	0.7349	0.5714	0.043*	
C1	0.6048 (2)	0.8506 (2)	0.3430 (2)	0.0292 (4)	
C2	0.6555 (3)	0.8776 (2)	0.1924 (2)	0.0331 (5)	
C3	0.1656 (3)	0.7297 (3)	0.6568 (2)	0.0411 (5)	
H3B	0.0955	0.7694	0.7185	0.049*	
H3C	0.1396	0.6294	0.6971	0.049*	
C4	0.1320 (2)	0.7852 (2)	0.5313 (2)	0.0351 (5)	
C5	0.0480 (3)	0.6995 (3)	0.5043 (2)	0.0449 (6)	
H5A	0.0178	0.6069	0.5614	0.054*	
C6	0.0088 (3)	0.7505 (3)	0.3932 (3)	0.0522 (7)	
H6A	−0.0472	0.6921	0.3767	0.063*	
C7	0.0533 (3)	0.8878 (3)	0.3073 (3)	0.0513 (7)	
H7A	0.0264	0.9224	0.2334	0.062*	
C8	0.1382 (3)	0.9738 (3)	0.3321 (3)	0.0502 (6)	
H8A	0.1690	1.0661	0.2742	0.060*	
C9	0.1776 (3)	0.9228 (3)	0.4432 (2)	0.0434 (6)	
H9A	0.2350	0.9813	0.4585	0.052*	
C10	0.5851 (3)	0.7012 (2)	0.7652 (2)	0.0363 (5)	
H10A	0.4726	0.6775	0.8196	0.044*	
H10B	0.6130	0.7932	0.7587	0.044*	
C11	0.6781 (2)	0.5975 (2)	0.8338 (2)	0.0302 (4)	
C12	0.7278 (3)	0.4795 (2)	0.8055 (2)	0.0358 (5)	
H12A	0.7076	0.4642	0.7395	0.043*	
C13	0.8075 (3)	0.3841 (2)	0.8745 (2)	0.0404 (5)	
H13A	0.8404	0.3058	0.8542	0.049*	
C14	0.8382 (3)	0.4048 (2)	0.9736 (2)	0.0423 (5)	
H14A	0.8918	0.3409	1.0195	0.051*	
C15	0.7883 (3)	0.5215 (3)	1.0035 (2)	0.0433 (6)	
H15A	0.8075	0.5355	1.0704	0.052*	
C16	0.7100 (3)	0.6174 (2)	0.9342 (2)	0.0371 (5)	
H16A	0.6780	0.6959	0.9544	0.045*	

### Atomic displacement parameters (Å^2^)

**Table:**

	*U*^11^	*U*^22^	*U*^33^	*U*^12^	*U*^13^	*U*^23^
Cl1	0.0426 (3)	0.0533 (4)	0.0418 (3)	0.0090 (3)	−0.0124 (3)	−0.0263 (3)
Cl2	0.0497 (4)	0.0798 (5)	0.0460 (4)	0.0072 (3)	−0.0276 (3)	−0.0271 (3)
Cl3	0.1147 (7)	0.0268 (3)	0.0370 (3)	−0.0035 (3)	−0.0111 (4)	−0.0010 (3)
P1	0.0396 (3)	0.0197 (3)	0.0240 (3)	0.0032 (2)	−0.0134 (2)	−0.0091 (2)
O1	0.0658 (11)	0.0215 (7)	0.0291 (8)	−0.0007 (7)	−0.0131 (7)	−0.0099 (6)
O2	0.0678 (11)	0.0236 (8)	0.0377 (9)	−0.0044 (7)	−0.0141 (8)	−0.0151 (7)
N1	0.0488 (10)	0.0193 (8)	0.0246 (9)	0.0026 (7)	−0.0129 (8)	−0.0107 (7)
N2	0.0368 (9)	0.0262 (9)	0.0341 (9)	0.0033 (7)	−0.0160 (8)	−0.0152 (7)
N3	0.0347 (10)	0.0469 (11)	0.0271 (9)	0.0040 (8)	−0.0122 (8)	−0.0135 (8)
C1	0.0365 (11)	0.0224 (10)	0.0286 (10)	0.0015 (8)	−0.0121 (9)	−0.0098 (8)
C2	0.0457 (12)	0.0254 (10)	0.0264 (10)	0.0015 (9)	−0.0130 (9)	−0.0083 (8)
C3	0.0354 (12)	0.0466 (13)	0.0325 (12)	−0.0013 (10)	−0.0095 (9)	−0.0083 (10)
C4	0.0267 (10)	0.0426 (12)	0.0342 (11)	0.0029 (9)	−0.0092 (9)	−0.0147 (10)
C5	0.0396 (12)	0.0473 (14)	0.0432 (13)	−0.0063 (10)	−0.0106 (11)	−0.0157 (11)
C6	0.0446 (14)	0.0704 (18)	0.0490 (15)	−0.0061 (12)	−0.0166 (12)	−0.0298 (14)
C7	0.0426 (13)	0.0755 (19)	0.0417 (14)	0.0074 (13)	−0.0215 (11)	−0.0230 (13)
C8	0.0509 (15)	0.0487 (15)	0.0487 (15)	0.0046 (12)	−0.0256 (12)	−0.0091 (12)
C9	0.0460 (13)	0.0406 (13)	0.0484 (14)	0.0012 (10)	−0.0255 (11)	−0.0138 (11)
C10	0.0413 (12)	0.0419 (12)	0.0343 (12)	0.0056 (10)	−0.0176 (10)	−0.0205 (10)
C11	0.0308 (10)	0.0335 (11)	0.0243 (10)	−0.0047 (8)	−0.0079 (8)	−0.0104 (8)
C12	0.0470 (12)	0.0352 (11)	0.0259 (10)	−0.0025 (9)	−0.0126 (9)	−0.0126 (9)
C13	0.0503 (13)	0.0304 (11)	0.0333 (12)	0.0023 (10)	−0.0104 (10)	−0.0096 (9)
C14	0.0462 (13)	0.0383 (13)	0.0347 (12)	0.0011 (10)	−0.0177 (10)	−0.0029 (10)
C15	0.0568 (14)	0.0461 (13)	0.0322 (12)	−0.0040 (11)	−0.0239 (11)	−0.0118 (10)
C16	0.0465 (13)	0.0384 (12)	0.0323 (11)	0.0004 (10)	−0.0166 (10)	−0.0174 (10)

### Geometric parameters (Å, °)

**Table:**

Cl1—C2	1.775 (2)	C6—C7	1.381 (4)
Cl2—C2	1.775 (2)	C6—H6A	0.9300
Cl3—C2	1.763 (2)	C7—C8	1.388 (4)
P1—O1	1.4787 (15)	C7—H7A	0.9300
P1—N2	1.6282 (17)	C8—C9	1.392 (4)
P1—N3	1.6296 (19)	C8—H8A	0.9300
P1—N1	1.7055 (17)	C9—H9A	0.9300
O2—C1	1.213 (2)	C10—C11	1.514 (3)
N1—C1	1.354 (3)	C10—H10A	0.9700
N1—H1	0.8600	C10—H10B	0.9700
N2—C3	1.472 (3)	C11—C12	1.389 (3)
N2—H2	0.8600	C11—C16	1.404 (3)
N3—C10	1.469 (3)	C12—C13	1.389 (3)
N3—H3	0.8600	C12—H12A	0.9300
C1—C2	1.570 (3)	C13—C14	1.387 (3)
C3—C4	1.516 (3)	C13—H13A	0.9300
C3—H3B	0.9700	C14—C15	1.385 (4)
C3—H3C	0.9700	C14—H14A	0.9300
C4—C9	1.391 (3)	C15—C16	1.384 (3)
C4—C5	1.398 (3)	C15—H15A	0.9300
C5—C6	1.391 (4)	C16—H16A	0.9300
C5—H5A	0.9300		
			
O1—P1—N2	111.32 (10)	C7—C6—C5	120.0 (2)
O1—P1—N3	119.71 (10)	C7—C6—H6A	120.0
N2—P1—N3	104.09 (9)	C5—C6—H6A	120.0
O1—P1—N1	106.89 (8)	C6—C7—C8	119.5 (2)
N2—P1—N1	112.12 (9)	C6—C7—H7A	120.2
N3—P1—N1	102.49 (9)	C8—C7—H7A	120.2
C1—N1—P1	123.22 (14)	C7—C8—C9	120.5 (3)
C1—N1—H1	118.4	C7—C8—H8A	119.8
P1—N1—H1	118.4	C9—C8—H8A	119.8
C3—N2—P1	123.37 (15)	C4—C9—C8	120.6 (2)
C3—N2—H2	118.3	C4—C9—H9A	119.7
P1—N2—H2	118.3	C8—C9—H9A	119.7
C10—N3—P1	123.29 (15)	N3—C10—C11	114.51 (18)
C10—N3—H3	118.4	N3—C10—H10A	108.6
P1—N3—H3	118.4	C11—C10—H10A	108.6
O2—C1—N1	124.99 (19)	N3—C10—H10B	108.6
O2—C1—C2	120.05 (18)	C11—C10—H10B	108.6
N1—C1—C2	114.96 (17)	H10A—C10—H10B	107.6
C1—C2—Cl3	109.68 (14)	C12—C11—C16	118.3 (2)
C1—C2—Cl1	109.66 (14)	C12—C11—C10	122.64 (18)
Cl3—C2—Cl1	109.28 (12)	C16—C11—C10	119.02 (19)
C1—C2—Cl2	109.45 (15)	C11—C12—C13	120.7 (2)
Cl3—C2—Cl2	109.43 (12)	C11—C12—H12A	119.6
Cl1—C2—Cl2	109.32 (11)	C13—C12—H12A	119.6
N2—C3—C4	114.92 (18)	C14—C13—C12	120.5 (2)
N2—C3—H3B	108.5	C14—C13—H13A	119.8
C4—C3—H3B	108.5	C12—C13—H13A	119.8
N2—C3—H3C	108.5	C15—C14—C13	119.4 (2)
C4—C3—H3C	108.5	C15—C14—H14A	120.3
H3B—C3—H3C	107.5	C13—C14—H14A	120.3
C9—C4—C5	118.2 (2)	C16—C15—C14	120.3 (2)
C9—C4—C3	121.5 (2)	C16—C15—H15A	119.8
C5—C4—C3	120.2 (2)	C14—C15—H15A	119.8
C6—C5—C4	121.1 (2)	C15—C16—C11	120.8 (2)
C6—C5—H5A	119.4	C15—C16—H16A	119.6
C4—C5—H5A	119.4	C11—C16—H16A	119.6

### Hydrogen-bond geometry (Å, °)

**Table:**

*D*—H···*A*	*D*—H	H···*A*	*D*···*A*	*D*—H···*A*
N1—H1···O1^i^	0.86	1.95	2.790 (2)	167
N2—H2···O2^ii^	0.86	2.23	3.055 (2)	161

Symmetry codes: (i) −*x*+1, −*y*+1, −*z*+1; (ii) −*x*+1, −*y*+2, −*z*+1.
